# γ-Mangostin attenuates osteoclastogenesis and bone resorption by suppressing the PI3K/AKT/NF-κB pathway

**DOI:** 10.3389/fphar.2025.1742401

**Published:** 2026-01-12

**Authors:** Jian Wei, Jiayue Xie, Zhiyang He, Xiaofeng Feng

**Affiliations:** Department of Joint Orthopedics, Liuzhou People’s Hospital Affiliated to Guangxi Medical University, Liuzhou, China

**Keywords:** bone resorption, osteoclastogenesis, PI3K/AKT/NF-κB pathway, postmenopausal osteoporosis, γ-Mangostin

## Abstract

**Background:**

Postmenopausal osteoporosis (PMOP), driven predominantly by estrogen deficiency-induced hyperactivation of osteoclasts, represents a critical public health burden. The pursuit of naturally sourced inhibitors of osteoclast function with minimized adverse effects remains a pivotal research endeavor. γ-Mangostin (γ-Mag), a natural xanthone derived from the pericarp of mangosteen, possesses broad anti-inflammatory and anti-tumor activities. Nevertheless, its influence on bone metabolic homeostasis, particularly osteoclast biology, remains entirely unexplored. This study aims to elucidate the impact of γ-Mag on osteoclast differentiation and function, and to evaluate its therapeutic potential for PMOP.

**Methods:**

Primary rat bone marrow-derived macrophages (BMMs) were isolated and stimulated with RANKL to establish an *in vitro* osteoclastogenesis model. The effects of γ-Mag on osteoclast formation and function were assessed through TRAP staining, F-actin ring immunofluorescence, and bone slice resorption pit assays. Mechanistic insights were gained by examining the PI3K/Akt/NF-κB pathway and downstream osteoclastogenic factors (C-FOS, NFATc1) using qRT-PCR, Western blot, and immunofluorescence. The *in vivo* efficacy was validated in an ovariectomized (OVX) rat model of PMOP, with bone microarchitecture and remodeling parameters analyzed *via* Micro-CT and bone histomorphometry.

**Results:**

At non-cytotoxic concentrations (≤4 μM), γ-Mag potently and concentration-dependently suppressed RANKL-induced osteoclast formation, disrupted F-actin ring integrity, and impaired bone resorptive activity. Mechanistically, γ-Mag significantly attenuated the RANKL-triggered activation of the PI3K/AKT/NF-κB signaling axis, as demonstrated by reduced phosphorylation of PI3K, AKT, p65, and IκB. This upstream suppression consequently led to the downregulation of the pivotal transcription factors C-FOS and NFATc1, and inhibited NFATc1 nuclear translocation. *In vivo*, γ-Mag administration (10 mg/kg, i.p., every other day for 8 weeks) markedly ameliorated bone loss and restored compromised bone microarchitecture in OVX rats, which was associated with reduced osteoclast numbers and decreased expression of *ACP5*, *CTSK*, *C-FOS*, and *NFATc1* in bone tissue.

**Conclusion:**

Our findings demonstrate that γ-Mag inhibits osteoclastogenesis and bone resorption by targeting the PI3K/AKT/NF-κB pathway, thereby blunting the C-FOS/NFATc1 transcriptional program. This study establishes γ-Mag as a promising natural lead compound for the treatment of postmenopausal osteoporosis.

## Introduction

Postmenopausal osteoporosis (PMOP) poses a significant global health burden for middle-aged and elderly women, primarily attributable to a precipitous decline in estrogen levels. The ensuing imbalance between bone resorption and formation culminates in reduced bone mass, deteriorated bone microarchitecture, and a substantially elevated fracture risk (Zhivodernikov et al., 2023). Osteoclasts, the sole multinucleated giant cells responsible for bone resorption, undergo excessive activation that represents a pivotal event in PMOP-related bone loss (Compston et al., 2019). Therefore, targeting and inhibiting osteoclast formation and function has emerged as a viable strategy for PMOP prevention and treatment (An et al., 2016).

The RANKL/RANK/OPG system serves as the principal pathway governing osteoclast differentiation. After RANKL binds to RANK on the membrane of osteoclast precursors, it recruits various adaptor proteins, thereby activating downstream signaling pathways including nuclear factor kappa-B (NF-κB), mitogen-activated protein kinase (MAPK), and phosphatidylinositol 3-kinase/protein kinase B (PI3K/Akt) (Boyle et al., 2003). Among these, the PI3K/Akt pathway not only modulates cell survival and proliferation but also acts synergistically with the NF-κB pathway to cooperatively activate the master regulator of osteoclast differentiation—nuclear factor of activated T cells c1 (NFATc1) (Moon et al., 2012). NFATc1 then initiates the expression of a suite of osteoclast-specific genes (such as Tartrate-resistant acid phosphatase Acp5, Cathepsin K CtsK, Matrix metalloproteinase 9 MMP9, etc.), ultimately driving osteoclast terminal differentiation and the execution of bone resorption function (Takayanagi, 2007). Consequently, targeting the PI3K/Akt/NF-κB axis offers a solid theoretical foundation for developing anti-osteoporosis drugs (Xu et al., 2023).

In recent years, the search for highly effective and low-toxicity lead compounds from natural products has gained considerable momentum (Whelan et al., 2006). γ-Mangostin (γ-Mag), a predominant active component isolated from the pericarp of the mangosteen fruit (Garcinia mangostana, Clusiaceae), belongs to the xanthone class of compounds. Substantial evidence from numerous studies indicates that γ-Mag possesses remarkable anti-inflammatory, antioxidant, antitumor, and neuroprotective effects (Chiu et al., 2020). Its diverse pharmacological mechanisms involve the regulation of multiple signaling pathways; for instance, it can induce apoptosis in cancer cells by inhibiting the PI3K/Akt/mTOR pathway (Choi et al., 2015) and alleviate inflammatory responses in models of inflammation by blocking the NF-κB pathway (Bumrungpert et al., 2009). Notably, while γ-Mag has been extensively studied for its anti-inflammatory and anti-cancer properties, its direct effects on bone metabolism remain largely unexplored. A limited number of studies have suggested that other xanthones, such as α-mangostin, may influence bone remodeling (Tantra et al., 2023; Zhang et al., 2022), but specific data concerning γ-Mag are scarce. For instance, one study reported that α-mangostin suppressed osteoclastogenesis via modulation of RANKL signaling (Zhang et al., 2022), yet the role of γ-Mag—a structurally distinct analog—remains elusive. This knowledge gap underscores the novelty and significance of our present investigation.

Based on this premise, our study proposes the scientific hypothesis: γ-Mag may inhibit osteoclast differentiation and function by interfering with the PI3K/Akt/NF-κB signaling pathway, thereby alleviating estrogen deficiency-induced osteoporosis. Elucidating the inhibitory effects of γ-Mag on osteoclasts not only addresses a critical research gap but also highlights its potential as a multi-target therapeutic agent for PMOP. Given its dual inhibitory action on both PI3K/Akt and NF-κB pathways—key drivers of osteoclast activation—γ-Mag may offer a superior therapeutic profile compared to single-target agents. Moreover, as a natural compound with established anti-inflammatory properties, γ-Mag could prove particularly beneficial for patients with inflammatory bone disorders, such as rheumatoid arthritis–associated osteoporosis (Bumrungpert et al., 2009). We tested this hypothesis through a combination of systematic *in vitro* and *in vivo* experiments, aiming to provide a promising candidate drug and a theoretical foundation for PMOP management.

## Materials and methods

### Reagents and antibodies

**FIGURE 1:**
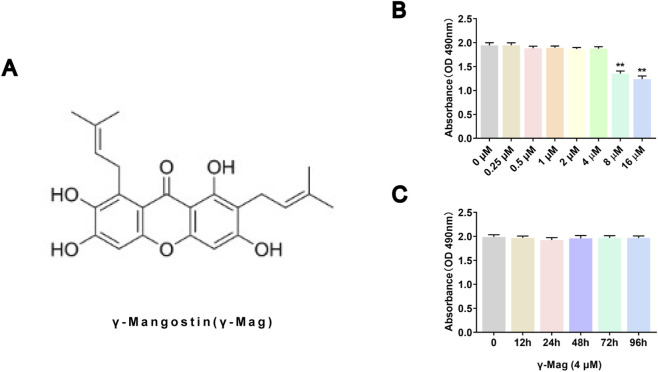
Cytotoxicity of γ-Mangostin (γ-Mag) in BMMs **(A)** Chemical structure of γ-Mag. **(B)** Viability of BMMs treated with increasing concentrations of γ-Mag for 48 h, assessed by MTS assay. **(C)** Viability of BMMs treated with 4 μM γ-Mag over time, measured by MTS assay. Data are presented as mean ± S.E.M. from three independent experiments, each performed with six replicates per group (n = 6). ^*^ *P* < 0.05, ^**^ *P* < 0.01 compared to the 0 μM group. One-way ANOVA followed by Tukey’s *post hoc* test was used for statistical analysis. OD, optical density; M-CSF, macrophage colony-stimulating factor; γ-Mag, γ-Mangostin; BMMs, bone marrow-derived macrophages; MTS, multiple tumor suppressor.

γ-Mangostin (purity ≥98%, HPLC, structure shown in Figure 1A) was purchased from Merck (United States, Catalog #: M6824), dissolved in dimethyl sulfoxide (DMSO) to prepare stock solutions, with the final concentration of DMSO in cell experiments not exceeding 0.1%. Recombinant rat M-CSF (Catalog #: HY-P7247, lot #: 508608) and RANKL (Catalog #: 9366-TN-025, lot #: DEIZ0322121) were purchased respectively from Medchemexpress Corporation and R&D Systems (United States). α-MEM medium, fetal bovine serum (FBS), and penicillin-streptomycin solution were purchased from Gibco (United States). TRAP staining kit, FITC-labeled phalloidin, and DAPI staining solution were purchased from Beyotime Biotechnology (China). TRIzol reagent was acquired from Invitrogen (United States). Primary antibodies for Western blot (PI3K, p-PI3K, AKT, p-AKT, P65, p-P65, IκB, p-IκB, C-FOS, NFATc1, CTSK, GAPDH) and corresponding HRP-conjugated secondary antibodies were purchased from Proteintech (China). Secondary antibodies for immunofluorescence were obtained from the same company.

### *In vitro* osteoclast induction and culture

All animal experimental procedures received approval from the Animal Ethics Committee of Liuzhou People’s Hospital. Four-week-old Wistar rats were used for BMM isolation to maintain species consistency with the *in vivo* osteoporosis model, thereby minimizing inter-species variability in the translational interpretation of results. Femora and tibiae were aseptically isolated. The bone marrow cavity was flushed with PBS containing 1% penicillin-streptomycin to collect bone marrow cell suspensions. After treatment with red blood cell lysis buffer, cells were resuspended in complete α-MEM medium supplemented with 10% FBS, seeded at an appropriate density, and cultured in medium containing 100 ng/mL M-CSF for 48 h. Non-adherent cells were discarded, and the adherent bone marrow-derived macrophages (BMMs) were harvested as osteoclast precursors. To induce osteoclast differentiation, BMMs were seeded at an appropriate density and cultured in medium containing 100 ng/mL M-CSF and 50 ng/mL RANKL, with medium changed every 2 days, and different concentrations of γ-Mag were added according to the experimental design.

### Cell viability assay (MTS)

BMMs were seeded in 96-well plates (2 × 10^4^ cells/well). After adherence, the medium was replaced with medium containing different concentrations of γ-Mag (0, 0.25, 0.5, 1, 2, 4, 8, 16 μM). After 48 h of culture, 20 μL of MTS solution (Promega, United States) was added to each well, followed by incubation for another 2 h. The absorbance (OD value) was measured at 490 nm using a microplate reader (BioTek, United States) to calculate relative cell viability. Then, the optimal concentration of γ-Mag was selected to intervene for 96 h, and cell viability was detected at 0, 12 h, 24 h, 48 h, and 96 h using the same method.

### TRAP staining

After 5 days of induction with RANKL, cells were fixed with 4% paraformaldehyde and then processed according to the TRAP staining kit instructions. After incubation at 37 °C in the dark for 60 min, cells were washed with PBS and counterstained with hematoxylin. TRAP-positive multinucleated cells (nuclei ≥3) were identified as mature osteoclasts and counted under an optical microscope (Nikon, Japan).

### F-actin immunofluorescence staining and bone resorption pit analysis

To observe actin rings, BMMs were seeded on 48-well plates. After induced differentiation, cells were fixed, permeabilized, and stained with FITC-phalloidin and DAPI for F-actin and nuclei, respectively, and observed under a fluorescence microscope. To assess bone resorption function, BMMs were seeded on sterile bovine bone slices and induced for 7 days. After cell removal, bone slices were immersed in 10% sodium hypochlorite solution to remove residual organic components, washed with distilled water, dried, and observed under a scanning electron microscope (Zeiss, Germany). The resorption pit area was quantified using ImageJ software.

### RNA extraction and quantitative real-time PCR (qRT-PCR)

Total RNA was extracted from cells or bone tissue using the TRIzol method. One microgram of RNA was reverse transcribed into cDNA using M-MLV reverse transcriptase (Promega, United States). Using cDNA as template, amplification was performed on a QuantStudio real-time PCR system (Applied Biosystems, United States) using SYBR Green Master Mix. The relative mRNA expression levels of target genes (*ACP5, CTSK, MMP9, C-FOS, NFATc1*) were calculated using the 2^−ΔΔCT^ method with *GAPDH* as the internal reference gene. Based on the established kinetics of osteoclastogenesis (Asagiri and Takayanagi, 2007; Boyce, 2013), cells were harvested at strategically selected time points to capture key transcriptional events: *C-FOS* mRNA at 24 h, *NFATc1* mRNA at 48 h, and osteoclast functional genes (*ACP5, CTSK, MMP9*) after 5 days of RANKL stimulation. The primer sequences are listed in Supplementary Table 1.

### Western blot analysis

Total protein was extracted from cells using RIPA lysis buffer (containing PMSF and phosphatase inhibitors). Protein concentration was determined by the BCA method. Equal amounts of protein were separated by SDS-PAGE and transferred to PVDF membranes. Membranes were blocked with 5% skim milk for 1 h, then incubated with specific primary antibodies (1:1000) at 4 °C overnight. After washing with TBST, membranes were incubated with HRP-conjugated secondary antibodies (1:5000) at room temperature for 1 h. Bands were visualized using ECL chemiluminescence reagent, and band intensity was analyzed using ImageJ software. For the analysis of PI3K/AKT/NF-κB pathway phosphorylation, cells were pre-treated with or without γ-Mag for 2 h, followed by stimulation with RANKL (50 ng/mL). Protein samples were harvested at the time points of peak phosphorylation for each protein, as determined by preliminary kinetic experiments: 15 min for p-PI3K and p-AKT, and 30 min for p-IκB and p-p65. For the analysis of CTSK, c-FOS, and NFATc1 protein expression, cells were harvested after 4 days of RANKL induction.

### Immunofluorescence analysis

Cell or decalcified bone paraffin sections were fixed, permeabilized, and blocked with serum. They were then incubated with primary antibodies (CTSK, NFATc1, 1:100) at 4 °C overnight. After PBS washing, they were incubated with Cy3 or FITC-labeled secondary antibodies (1:200) at room temperature in the dark for 1 h. Nuclei were stained with DAPI before mounting. Observations and photography were performed under a fluorescence microscope, and the mean fluorescence intensity was analyzed using ImageJ software. For *in vitro* studies, cells were fixed and stained after 4 days of RANKL induction for CTSK and NFATc1 analysis. For NFATc1 nuclear translocation studies, cells were stimulated with RANKL for 48 h. For *in vivo* studies, decalcified bone paraffin sections from the end of the 8-week experiment were used.

### Animal experiment design

Thirty 8-week-old female Wistar rats were randomly divided into 3 groups (n = 10): (1) Sham operation group (Sham group, intraperitoneal injection of saline); (2) OVX model group (OVX group, intraperitoneal injection of saline); (3) OVX + γ-Mag group (γ-Mag, 10 mg/kg, intraperitoneal injection, once every other day). This dosage was selected based on preliminary dose-finding experiments and supported by previous *in vivo* pharmacological studies of γ-Mangostin reporting efficacy and safety at comparable doses (e.g., 10–40 mg/kg) (Chiu et al., 2020; Khayat et al., 2023; Wang et al., 2019). After 1 week of acclimatization feeding, all rats underwent bilateral ovariectomy (Sham group had ovaries exposed and a portion of periovarian fat removed before suturing). Drug administration via intraperitoneal injection began 2 week post-surgery and continued for 8 weeks. At the end of the experiment, serum and femur samples were collected.

### Animal ethics and euthanasia

At the end of the experiment, euthanasia was humanely performed to collect tissue samples. Briefly, mice were deeply anesthetized with an intraperitoneal injection of xylazine (10 mg/kg) and ketamine (100 mg/kg). Upon confirming the absence of a pedal reflex, a lethal dose of sodium pentobarbital (150 mg/kg) was administered intraperitoneally to ensure death, which was verified by the cessation of both respiration and heartbeat. This protocol strictly adhered to the AVMA Guidelines for the Euthanasia of Animals (2020) and received approval from the Institutional Animal Care and Use Committee (IACUC).

### Micro-CT analysis

The right femora of rats were collected and scanned using a Skyscan1276 micro-CT system (Bruker, Germany) focusing on the distal femoral metaphysis. Scanning parameters: voltage 85 kV, current 200 μA, resolution 17.4 μm. Three-dimensional reconstruction was performed using supporting software, and bone volume fraction (BV/TV), trabecular number (Tb.N), trabecular thickness (Tb.Th), and trabecular separation (Tb.Sp) were analyzed.

### Bone histomorphometry

Femur samples were fixed in 4% paraformaldehyde for 48 h and decalcified in 10% EDTA for 4 weeks. After paraffin embedding, 4 μm thick sections were prepared for H&E staining and TRAP staining. An image analysis system was used to measure the percentage of osteoclast surface per bone surface (Oc.S/BS) and count the number of osteoclasts per unit bone perimeter.

### Statistical analysis

All data are presented as mean ± standard error of the mean (S.E.M.). For all cell-based assays, experiments were performed in triplicate wells per condition and repeated independently at least three times. Data are presented as mean ± S.E.M. from biological replicates (n ≥ 3). Statistical analysis was performed using GraphPad Prism 8.0 software. Comparisons among multiple groups were performed using one-way analysis of variance (ANOVA), and pairwise comparisons between groups were conducted using Tukey’s *post hoc* test. A *P* value <0.05 was considered statistically significant.

## Results

### γ-Mag inhibits osteoclast formation and function without cytotoxicity

**FIGURE 2:**
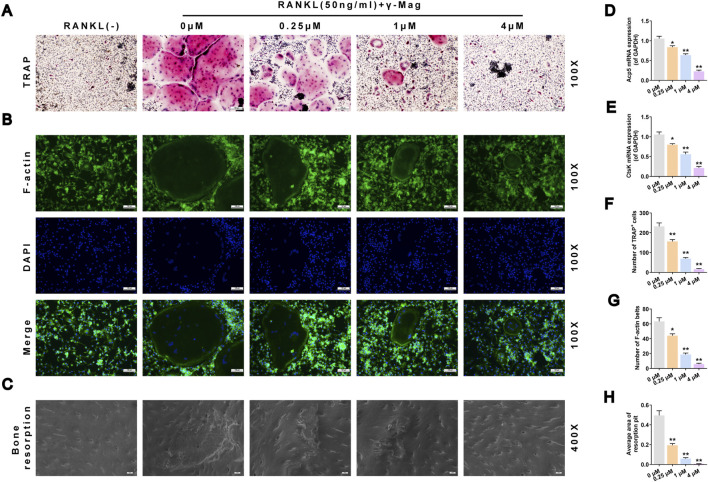
γ-Mag inhibits osteoclast differentiation, cytoskeletal formation, and bone resorption. **(A)** Representative images of TRAP-stained osteoclasts (red). **(B)** Fluorescence images of F-actin rings (green) and nuclei (blue, DAPI). **(C)** Scanning electron micrographs of resorption pits on bone slices. **(D,E)** Relative mRNA expression levels of **(D)** *ACP5* and **(E)** *CTSK* in osteoclasts treated with γ-Mag. **(F–H)** Quantitative analyses of **(F)** the number of TRAP-positive multinucleated cells, **(G)** the number of F-actin rings, and **(H)** the resorption pit area relative to total area. Data are shown as mean ± S.E.M. from three independent experiments, each performed with triplicate wells per condition (for TRAP, F-actin, resorption pit) or six replicates (for qRT-PCR). ^*^ *P* < 0.05, ^**^ *P* < 0.01 versus the 0 μM group. One-way ANOVA with *Tukey’s* test was applied. TRAP, tartrate-resistant acid phosphatase; *ACP5*, acid phosphatase 5; *CTSK*, cathepsin K; γ-Mag, γ-Mangostin.

MTS assay results showed that γ-Mag at concentrations ≤4 μM had no significant effect on the proliferation viability of BMMs (*P* > 0.05, Figure 1B). At 4 μM, γ-Mag also showed no obvious toxicity at different time points (12–96 h) (*P* > 0.05, Figure 1C). In functional experiments, TRAP staining showed that γ-Mag concentration-dependently reduced the number of RANKL-induced mature osteoclasts (*P* < 0.05, *P* < 0.01, Figures 2A,F). F-actin immunofluorescence revealed that γ-Mag treatment significantly disrupted the characteristic sealing zone structure of osteoclasts, with both the number and area of F-actin rings significantly decreased (*P* < 0.05, *P* < 0.01, Figures 2B,G). γ-Mag also suppressed the expression of osteoclast marker genes *ACP5* and *CTSK* (*P* < 0.05, *P* < 0.01, Figures 2D,E). Further bone slice resorption assays confirmed that the bone resorption pit area in the γ-Mag treated group was much smaller than that in the positive control group (*P* < 0.01, Figures 2C,H). These results indicate that γ-Mag effectively inhibits osteoclast differentiation, maturation, and bone resorption function at non-toxic concentrations.

### γ-Mag acts by inhibiting the PI3K/AKT/NF-κB pathway and its downstream signaling cascade

**FIGURE 3:**
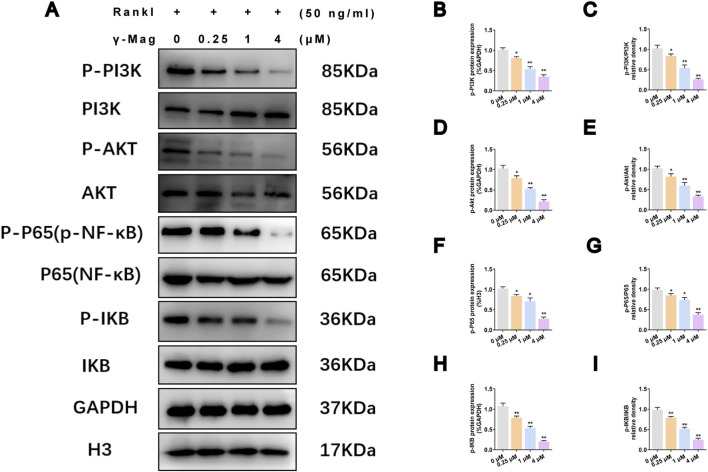
γ-Mag suppresses the PI3K/AKT/NF-κB pathway in osteoclasts. **(A)** Representative western blots of phosphorylated and total proteins of PI3K, AKT, P65 (NF-κB), and IκB in γ-Mag-treated osteoclasts. Cells were pre-treated with γ-Mag for 2 h and stimulated with RANKL for 15 min (for p-PI3K and p-AKT) or 30 min (for p-IκB and p-p65). **(B–I)** Densitometric quantification of protein levels expressed as phosphorylated/total ratios for **(B,C)** PI3K, **(D,E)** AKT, **(F,G)** P65, and **(H,I)** IκB. Data represent mean ± S.E.M. of three independent experiments (n = 3). ^*^ *P* < 0.05, ^**^ *P* < 0.01 compared to the 0 μM group. One-way ANOVA with *Tukey’s* test was used. PI3K, phosphatidylinositol 3-kinase; p-Akt, phosphorylated protein kinase B; γ-Mag, γ-Mangostin.

To elucidate the molecular mechanism, we first examined the effect of γ-Mag on the PI3K/Akt/NF-κB pathway. Western blot results showed that γ-Mag treatment concentration-dependently reduced the RANKL-induced phosphorylation levels of PI3K, AKT, P65, and IκB (*P* < 0.05, *P* < 0.01, Figures 3A–I), without affecting total protein levels, indicating specific inhibition of the activation of this signaling axis.

**FIGURE 4:**
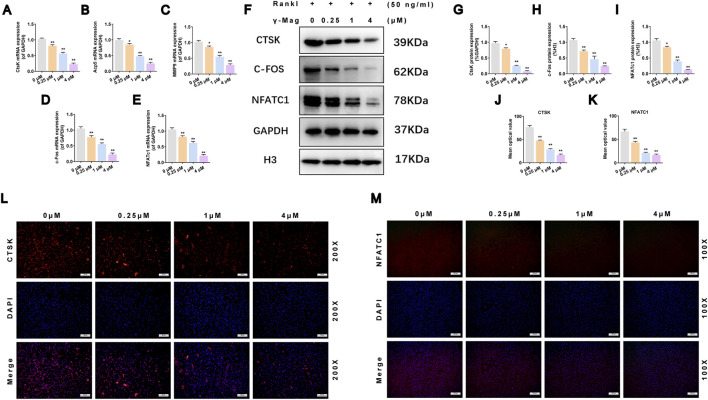
γ-Mag downregulates osteoclastogenic markers and transcription factors **(A–E)** qRT-PCR analysis of mRNA expression for **(A)** *CTSK*, **(B)** *ACP5*, **(C)** *MMP9*, **(D)** *C-FOS*, and **(E)** *NFATc1*. **(F)** Representative western blots of CTSK, C-FOS, and NFATc1 protein expression. The appearance of two bands for NFATc1 is attributed to the polyclonal antibody recognizing multiple isoforms; both bands were included in the densitometric analysis. **(G–I)** Quantification of protein levels for **(G)** CTSK, **(H)** C-FOS, and **(I)** NFATc1. **(J,K)** Mean fluorescence intensity of **(J)** CTSK and **(K)** NFATc1. **(L)** Immunofluorescence images of CTSK (red) and nuclei (blue, DAPI). **(M)** Immunofluorescence images of NFATc1 (red) and nuclei (blue, DAPI). Data are expressed as mean ± S.E.M. from three independent experiments (Western blot, immunofluorescence) or six replicates from three independent experiments (qRT-PCR). ^*^ *P* < 0.05, ^**^ *P* < 0.01 versus the 0 μM group. One-way ANOVA with Tukey’s test was performed. C-FOS, proto-oncogene C-FOS; NFATc1, nuclear factor of activated T cells 1; CTSK, cathepsin K; γ-Mag, γ-Mangostin.

Subsequently, we investigated downstream key transcriptional events. qRT-PCR results showed that γ-Mag significantly downregulated the mRNA expression of osteoclast functional genes (*ACP5, CTSK, MMP9*) and core transcription factors (*C-FOS, NFATc1*) (*P* < 0.05, *P* < 0.01, Figures 4A–E). At the protein level, Western blot analysis similarly showed significant inhibition of CTSK, C-FOS, and NFATc1 protein expression (*P* < 0.05, *P* < 0.01, Figures 4F–I). The appearance of two bands for NFATc1 is attributed to the polyclonal antibody recognizing multiple isoforms; both bands were included in the densitometric analysis. Immunofluorescence staining further visually confirmed that γ-Mag treatment reduced the fluorescence intensity of CTSK within osteoclasts (Figures 4J,L) and effectively blocked the nuclear translocation of NFATc1 (Figures 4K,M). These data collectively outline γ-Mag’s action pathway: by inhibiting the upstream PI3K/AKT/NF-κB signal, it subsequently interferes with the activation of the C-FOS/NFATc1 transcriptional axis, ultimately curbing osteoclast terminal differentiation.

### γ-Mag alleviates bone loss in OVX rats

**FIGURE 5:**
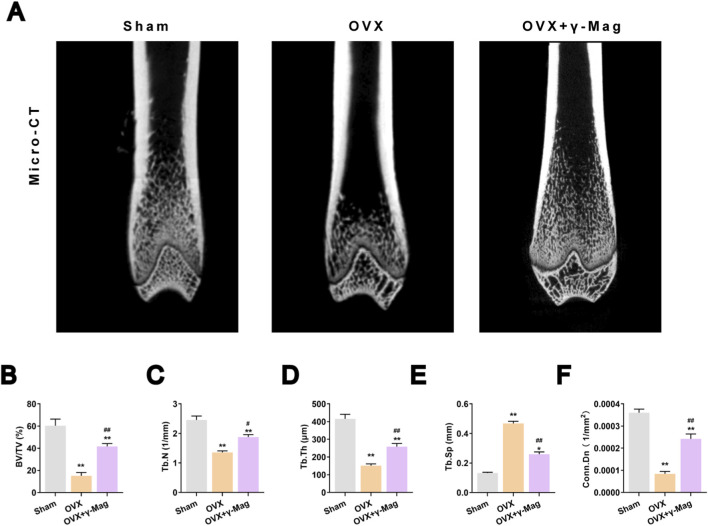
γ-Mag ameliorates OVX-induced bone loss in rats. **(A)** Representative micro-CT reconstructions of distal femurs from Sham, OVX,and OVX+γ-Mag groups. **(B–F)** Quantitative analysis of femoral microarchitecture: **(B)** BV/TV, **(C)** Tb.N, **(D)** Tb.Th, **(E)** Tb.Sp, and **(F)** Conn.Dn. Data are presented as mean ± S.E.M. (n = 10 rats/group). ^*^ *P* < 0.05, ^**^ *P* < 0.01 (vs. Sham group); ^##^ *P* < 0.05, ^##^ *P* < 0.01 (vs. OVX group). One-way ANOVA with *Tukey’s* test was applied. OVX, ovariectomized; γ-Mag, γ-Mangostin; BV/TV, bone volume/tissue volume; Tb.N, trabecular number; Tb.Th, trabecular thickness; Tb.Sp, trabecular separation; Conn.Dn, connectivity density.

The *in vivo* efficacy of γ-Mag was validated using the OVX rat model. Micro-CT three-dimensional reconstruction showed sparse and fractured trabeculae in the distal femur of OVX group rats, whereas the trabecular structure was noticeably repaired in the γ-Mag treatment group (Figure 5A). Quantitative analysis demonstrated that compared to the OVX group, γ-Mag treatment significantly increased bone volume fraction (BV/TV), trabecular number (Tb.N), trabecular thickness (Tb.Th), and trabecular connectivity density (Conn.Dn), while decreasing trabecular separation (Tb.Sp) (*P* < 0.05, *P* < 0.01, Figures 5B–E), with effects approaching those of the Sham group.

### γ-Mag reduces osteoclast activity *in vivo* by suppressing the C-FOS/NFATc1 pathway

**FIGURE 6:**
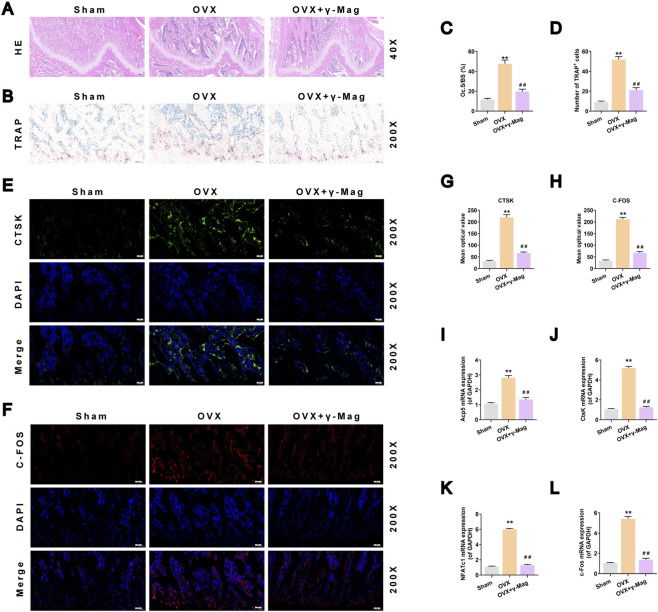
γ-Mag suppresses osteoclast activity and bone loss via c-FOS/NFATc1 downregulation in OVX rats **(A)** H&E-stained sections of decalcified distal femur (40X). **(B)** TRAP-stained sections of decalcified distal femur (200X). **(C,D)** Histomorphometric analysis of **(C)** Oc.S/BS and **(D)** N.Oc/B.Pm. **(E,F)** Immunofluorescence staining of bone sections for **(E)** CTSK (green) and **(F)** c-FOS (red) with DAPI (blue). **(G,H)** Mean fluorescence intensity of **(G)** CTSK and **(H)** c-FOS. **(I-L)** qRT-PCR analysis of mRNA expression in bone tissue for **(I)** Acp5, **(J)** CTSK, **(K)** Nfatc1, and **(L)** c-Fos. Data are shown as mean ± S.E.M. (n = 5 rats/group). ^*^ *P* < 0.05, ^**^ *P* < 0.01 (vs. Sham group); ^##^ *P* < 0.05, ^##^ *P* < 0.01 (vs. OVX group). One-way ANOVA with *Tukey’s* test was used. OVX, ovariectomized; γ-Mag, γ-Mangostin; H&E, hematoxylin and eosin; TRAP, tartrate-resistant acid phosphatase; Oc.S/BS, osteoclast surface/bone surface; N.Oc/B.Pm, number of osteoclasts/bone perimeter; Acp5, acid phosphatase 5; CTSK, cathepsin K; c-Fos, proto-oncogene c-Fos; NFATc1, nuclear factor of activated T cells 1.

Histological analysis provided cellular evidence supporting the micro-CT results. H&E staining showed that γ-Mag treatment ameliorated the OVX-induced deterioration of trabecular structure and the Oc.S/BS ratio (*P* < 0.01, Figures 6A,C). TRAP staining and quantitative analysis indicated that γ-Mag significantly reduced the number of osteoclasts on the bone surface in OVX rats (*P* < 0.01, Figures 6B,D). For molecular mechanism validation, immunofluorescence of bone tissue showed markedly lower protein expression levels of CTSK and C-FOS in the γ-Mag group (Figures 6E–H). qRT-PCR results further confirmed that γ-Mag treatment significantly downregulated the mRNA expression of *ACP5, CTSK, C-FOS*, and *NFATc1* in bone tissue (*P* < 0.01, Figures 6I–L). These *in vivo* data strongly demonstrate that the bone-protective effect of γ-Mag is achieved by inhibiting the C-FOS/NFATc1 signaling pathway, thereby curbing excessive osteoclast activity.

## Discussion

**FIGURE 7:**
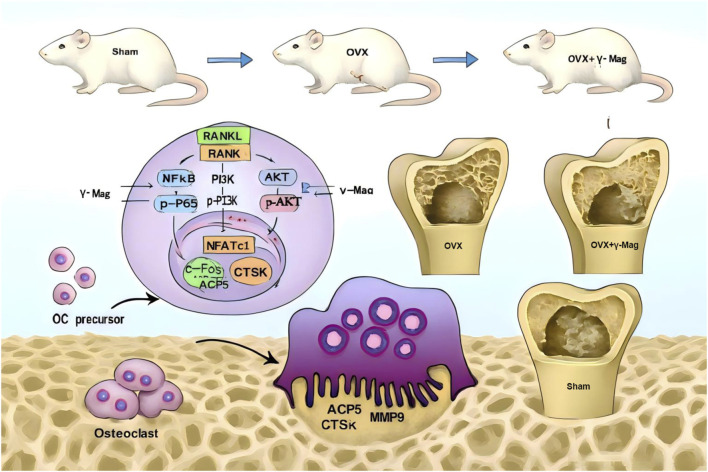
γ-Mangostin Attenuates Osteoclastogenesis and Prevents Ovariectomy-Induced Bone Loss via Suppressing the PI3K/Akt/NF-κB Signaling Pathway (A) γ-Mangostin exerts anti-osteoporotic effects *in vivo* by alleviating bone loss and improving bone microarchitecture in ovariectomized rats. *In vitro*, it suppresses RANKL-induced osteoclast formation, F-actin assembly, and bone resorption activity. The underlying mechanism involves the inhibition of the PI3K/AKT/NF-κB signaling pathway through downregulation of phosphorylation levels of PI3K, AKT, p65, and IκB. This leads to reduced expression of the downstream transcription factors c-Fos and NFATc1, as well as inhibition of NFATc1 nuclear translocation. Consequently, the expression of key osteoclast-related functional genes—ACP5, CTSK, and MMP9—is downregulated, ultimately attenuating osteoclast differentiation and bone resorption activity.

This study provides comprehensive evidence that γ-mangostin (γ-Mag), a natural xanthone derived from mangosteen, exerts potent anti-osteoporotic effects through dual inhibition of osteoclast differentiation and function. Notably, our findings extend beyond the mere documentation of these effects, revealing a sophisticated mechanistic pathway wherein γ-Mag concurrently attenuates the PI3K/AKT and NF-κB signaling axes. This coordinated suppression leads to the downstream disruption of the C-FOS/NFATc1 transcriptional cascade, ultimately reducing bone resorption and protecting against estrogen deficiency-induced bone loss in our OVX rat model (Figure 7). By delineating this dual-pathway inhibition, our work positions γ-Mag as a functionally distinct candidate amidst the landscape of natural products with anti-osteoclastogenic properties.

The pathological bone loss characteristic of postmenopausal osteoporosis (PMOP) is primarily driven by excessive osteoclast activity following estrogen withdrawal (Feng and McDonald, 2011). The RANKL/RANK signaling pathway serves as the master regulator of osteoclastogenesis, wherein the early activation of both PI3K/Akt and NF-κB pathways is critical for initiating the differentiation program (Liu F. et al., 2024; Moon et al., 2012). Our data demonstrate that γ-Mag uniquely and simultaneously targets these two pivotal pathways. This was evidenced by its significant inhibition of RANKL-induced phosphorylation of PI3K, AKT, IκB, and p65 (Figure 3). The pharmacological significance of this dual inhibition is substantial. Firstly, the suppression of the NF-κB pathway prevents the nuclear translocation of p65, thereby obstructing the transcription of early osteoclastogenic genes (Liu F. et al., 2024). Secondly, the concomitant inhibition of the PI3K/AKT pathway disrupts a crucial survival and proliferation signal for osteoclast precursors (Liu H. et al., 2024). This multi-target action aligns with the emerging and desirable paradigm in natural product drug discovery, where compounds exhibiting polypharmacological profiles often demonstrate enhanced efficacy and potentially superior therapeutic outcomes compared to single-target agents (Gupta et al., 2013).

When contextualizing our findings within the existing literature on natural anti-osteoclastogenic compounds, the distinct advantage of γ-Mag becomes apparent. For instance, Echinacoside has been reported to primarily target the PI3K/AKT axis to alleviate osteolysis (Jiang et al., 2022). In contrast, γ-Mag’s capacity to concurrently inhibit the NF-κB pathway suggests a broader and potentially more robust mechanistic scope. This characteristic may be particularly advantageous for treating inflammatory bone diseases, such as rheumatoid arthritis, where NF-κB serves as the primary mediator of inflammatory cytokine-induced osteoclastogenesis. Furthermore, comparison with other mangosteen xanthones highlights structure-activity nuances. While α-mangostin has been shown to suppress osteoclastogenesis via modulation of RANKL signaling (Zhang et al., 2022), our study is the first to elucidate the specific and dual inhibitory role of its structural analog, γ-Mag, on the PI3K/AKT/NF-κB axis. Another compelling parallel can be drawn with Neoandrographolide (NEO), a natural diterpenoid recently reported to inhibit osteoclast differentiation through multiple pathways, including MAPK and NF-κB, while also affecting the PI3K/AKT pathway (Tang et al., 2025). The observation that γ-Mag demonstrates a similarly multi-targeted efficacy, yet through a distinctly defined dual-pathway core involving PI3K/AKT and NF-κB, reinforces the therapeutic value of such a mechanism in achieving potent anti-osteoclastogenic effects.

The convergence point of multiple osteoclastogenic signals is the induction of the master transcription factors C-FOS and NFATc1 (Matsuo and Irie, 2008). NFATc1, in particular, initiates an auto-amplification loop that drives the expression of a suite of osteoclast-specific genes, such as *ACP5*, *CTSK*, and *MMP9*(Asagiri and Takayanagi, 2007; Lu et al., 2024). Our results convincingly show that γ-Mag’s upstream suppression of both PI3K/AKT and NF-κB signaling cascades translates into a profound downregulation of both C-FOS and NFATc1 at the transcriptional and protein levels. Crucially, our immunofluorescence data demonstrated that γ-Mag not only reduces NFATc1 expression but effectively prevents its nuclear translocation (Figure 4). This step is fundamental, as nuclear localization is absolutely essential for NFATc1’s transcriptional activity (Soe et al., 2021). By sequestering NFATc1 in the cytoplasm, γ-Mag fundamentally interrupts the terminal differentiation program, leading to the comprehensive suppression of osteoclast functional genes (Figures 2D,E, 4A–C) and a consequent impairment of bone resorptive capacity (Figures 2C,H).

The translational significance of our cellular findings was strongly validated in the OVX rat model, which faithfully recapitulates the high bone turnover state of human PMOP (Yousefzadeh et al., 2020). The 8-week γ-Mag treatment regimen significantly ameliorated OVX-induced bone loss and microarchitectural deterioration (Figure 5). More importantly, we obtained direct molecular evidence from bone tissue, demonstrating that the therapeutic efficacy correlated with reduced expression of C-FOS, NFATc1, and key osteoclast activity markers (Figure 6). This coherent data chain, spanning from molecular pathways to cellular function and ultimately to whole-bone phenotype, forms a robust translational bridge supporting γ-Mag’s potential therapeutic application.

Beyond its direct inhibitory effects on osteoclasts, recent research underscores the importance of the bone immune microenvironment and the interplay between bone resorption and formation (Tsukasaki and Takayanagi, 2019). γ-Mag’s documented anti-inflammatory properties (Bumrungpert et al., 2009; Liu F. et al., 2024) may provide additional benefits by modulating this crosstalk, potentially creating a more favorable microenvironment for bone remodeling. This prospect, while beyond the scope of the present study, opens an exciting avenue for future investigation.

### Limitations and future perspectives

While this study provides compelling evidence for γ-Mag as a potent inhibitor of osteoclastogenesis, we acknowledge several limitations that should be addressed in future work. Firstly, our research focused exclusively on the osteoclast aspect of bone remodeling. A complete assessment of γ-Mag’s therapeutic potential for osteoporosis must necessarily investigate its effects on osteoblastic bone formation. Indeed, the net bone balance in osteoporosis is determined by the coupling between resorption and formation. Future studies are warranted to determine whether γ-Mag influences osteogenic differentiation and bone formation, which would provide a more comprehensive understanding of its overall impact on bone metabolism (Park, 2025; Tan et al., 2025). Secondly, the animal model used, while classic for PMOP, does not fully capture the complexity of human disease. Future evaluations of γ-Mag in other osteolytic models, such as glucocorticoid-induced osteoporosis, would strengthen the case for its broader applicability. Furthermore, comprehensive pharmacokinetic studies, oral bioavailability assessment, and long-term safety profiling are essential next steps for advancing γ-Mag toward clinical development. Thirdly, Although this study focused on the mechanistic actions of γ-Mag, future work will include head-to-head comparisons with standard therapies (e.g., alendronate or denosumab) to better evaluate its relative efficacy and potential clinical advantages. Finally, given the emergence of natural products targeting bone metabolism, such as Yam polysaccharide reported to promote osteoblast differentiation via the CASP3/PI3K/AKT pathway (Yu et al., 2025), and 5-Methoxypsoralen, which targets the Notum/Wnt pathway to combat osteoporosis (Song et al., 2025), exploring potential synergistic effects between γ-Mag and such agents could lead to novel and powerful combination therapies for osteoporosis.

## Conclusion

In summary, this study elucidates that γ-mangostin, by coordinately targeting and inhibiting the PI3K/AKT/NF-κB signaling network, effectively blocks the RANKL-induced C-FOS/NFATc1 transcriptional cascade, thereby exhibiting significant anti-osteoclastogenic and anti-resorptive activity in both *in vitro* and *in vivo* models. The dual-pathway mechanism we uncovered not only distinguishes γ-Mag from many single-target natural compounds but also enhances its promise as a therapeutic agent. These findings not only reveal the great potential of γ-Mag as a novel natural lead compound against osteoporosis but also provide a solid scientific basis for the further development of new drugs based on it for the treatment of postmenopausal osteoporosis and related osteolytic diseases.

## Data Availability

The original contributions presented in the study are included in the article/Supplementary Material, further inquiries can be directed to the corresponding author.
