# Supplementary material for: γ-Mangostin attenuates osteoclastogenesis and bone resorption by suppressing the PI3K/AKT/NF-κB pathway
Source: Front Pharmacol. 2026 Jan 12;16:1742401. doi: 10.3389/fphar.2025.1742401 (PMC12833320; doi:10.3389/fphar.2025.1742401)
Supplement: Supplementary file 1 [file Table1.docx]

**Table 1. Primer sequences for qRT-PCR in this study.**

| **Target genes** | **Forward primers** | **Reverse primers** |
| --- | --- | --- |
| CTSK | TATGGAAAGAAGACTCACCAGAAAGC | GGTATTCTGAGTCCCGTCATCTTC |
| ACP5 | CACTCCACCCCTGAGATTTGT | CCCGAGACATGATGAAGTCA |
| MMP9 | ACTGACAAGAAGTGGGGGTTTCGT | GCTTAGAGCCACGACCATACAGA |
| C-FOS | AACCGCCACGATGATGTTCT | TCTGCGGGTGAGTGGTAGTA |
| NFATc1 | GGAGATCCGAGAA TCGAGAT | TTGCAGCAGGAAGTACGTCT |
| GAPDH | CCTCGTCCCGTAGACAAAATG | TGAGGTCAAATGAAGGGGTCGT |

ACP5: Acid phosphatase 5; CTSK: Cathepsin K; C-FOS: Protooncogene c-Fos; NFATc1: Nuclear factor of active T cells 1; MMP9: Matrix Metallopeptidase 9; GAPDH: glyceraldehyde-3-phosphate dehydrogenase.
